# Consumption of milk and milk products in the population of the Upper Silesian agglomeration inhabitants

**DOI:** 10.3402/fnr.v60.28976

**Published:** 2016-03-01

**Authors:** Marek Kardas, Elżbieta Grochowska-Niedworok, Beata Całyniuk, Ilona Kolasa, Mateusz Grajek, Agnieszka Bielaszka, Agata Kiciak, Małgorzata Muc-Wierzgoń

**Affiliations:** 1 Department of Food Technology and Quality Evaluation, Faculty of Public Health in Bytom, Medical University of Silesia, Zabrze, Poland; 2 Department of Human Nutrition, Faculty of Public Health in Bytom, Medical University of Silesia, Zabrze, Poland; 3 Department of Internal Diseases, Faculty of Public Health in Bytom, Medical University of Silesia, Zabrze, Poland

**Keywords:** milk, milk products, diet, nutrition

## Abstract

**Background:**

Providing the appropriate amount of nutrients at every stage of life is a key element determining the proper development and functioning of the body.

**Objective:**

Because of the nutritional value and resulting position of milk and milk products in the daily diet, this study was undertaken to assess the consumption of milk and milk products among the inhabitants of the Upper Silesian agglomeration.

**Design:**

The survey covered 600 people, including 339 women (56.5%) and 261 men (43.5%) aged 18–78 years. To assess the consumption of milk and milk products, as a research tool an original survey with the closed-ended and open-ended questions was used. The questions concerned the characteristics of the surveyed group and various aspects of the consumption of milk and milk products. The results obtained were subjected to statistical analysis using the Statistica 10.0 program with a chi-square test for quality features.

**Results:**

The level of consumption of milk and milk products among the Upper Silesian agglomeration inhabitants is insufficient in relation to nutrition recommendations. However, despite many controversies surrounding milk, the respondents also claimed that it played an important role in their daily diet.

**Conclusions:**

The most frequently consumed type of milk in the surveyed group is ultra heat treated (UHT) milk with average fat content.

Providing the appropriate amount of nutrients at every stage of life is a key element determining the proper development and functioning of the body. Nutrition standards, which determine the demand for nutrients depending on age, sex, and physical activity, as well as rational nutrition rules developed on the basis of these standards, define the proportions between the consumption of various food products, which are a source of different nutrients (1, 2).

Milk and its products play an important role in nutrition of human beings, particularly children and adolescents during the period of their intense growth and development. At the bottom of the healthy nutrition pyramid, there are cereal products, then vegetables, fruit, and milk and milk products (3). The high position of milk in various forms and in the general rules of human nutrition indicates the great importance of this product group in meeting the body's demand for nutrients. Dairy products provide a wide range of necessary building and regulating components (4).

Milk and dairy products are some of the foods that are rich in a whole range of vital constituents. The most important of these include calcium and vitamin D. The presence of iodine is also important. Its content in milk depends on its presence in the feed eaten by cows (5, 6). The iodine content in the milk on the Polish market indicates that the consumption of a 200-ml portion of milk meets 20–30% of the daily demand of the youth population and barely 13–20% of the demand for this constituent in adults (7). Given the fact that iodine deficiency poses serious health risks at the population level, in Poland iodine prophylaxis is implemented. It is based on the mandatory iodination of kitchen salt (30±10 g KJ/kg) and baby formulas. This solution, which has been in place since 1996, has turned out to be very effective; still, the latest World Health Organization (WHO) recommendations on the limitation of salt consumption as a risk factor in diseases of the vascular system make it necessary to introduce additional iodine carriers in food products, such as natural mineral water and milk (8).

Nutrition recommendations on the consumption levels of milk and dairy products in Poland are presented to consumers in the form of a food pyramid developed by experts from the National Food and Nutrition Institute in Warsaw (9). These recommendations correspond with the WHO recommendations. Moreover, an educational campaign has been carried out at the national level – with the message ‘Drink Milk and You Will Be Big!’

Currently, milk is increasingly being investigated by researchers in terms of its positive impact on human health and development. The latest scientific publications provide, on the one hand, substantial evidence to confirm the health benefits resulting from consumption of this product group and in particular fermented milk products (10, 11). However, on the other hand, some publications indicate the opposite effect, associated mainly with the consumption of milk (12). There are also opinions indicating a need to modify the recommendations on the consumption of milk and milk products, in particular among adults (13).

Given the above and the nutrition value and resulting position of milk and milk products in the daily diet, studies have been taken to assess the consumption of milk and milk products among the Upper Silesian agglomeration inhabitants.

## Materials and methods

The planned research was conducted in the first quarter of 2013 among the adult inhabitants of the Upper Silesian agglomeration. The area of the study is an important element of the population system of the European importance. It is a highly urbanised region, where more than 80% of the population live in cities. In addition, this area is characterised by the highest population rate in Poland. The study did not require the consent of the local ethics committee.

The method applied consisted of a diagnostic survey carried out using a proprietary survey questionnaire among 820 persons. The survey was distributed in paper form among voluntary participants. The individuals were recruited by non-random snowball sampling. The criteria for inclusion in the study included majority and a permanent place of residence in the Upper Silesian agglomeration. The exclusion criterion was the respondent's statement that he or she did not tolerate and/or was allergic to milk. In all, 600 persons correctly filled in the questionnaire, of whom 339 were women (56.5%) and 261 men (43.5%).

The survey questionnaire consisted of two parts. The first part included questions about age, gender, height, and body weight. On the basis of the data obtained, body mass index (BMI) was calculated (1). The other part contained questions about different aspects of the consumption of milk and dairy products. These questions included some concerning the frequency and type of the products consumed, as well as questions enabling an assessment of the respondents’ nutritional awareness of the importance of such products in their diet.

In order to demonstrate the dependencies between gender, age, and consumption frequency, the type of milk consumed, and respondents’ awareness, the results were subjected to a statistical analysis in the Statistica 10.0 program using a chi-square test for qualitative characteristics and the Yates and Fisher correction for small groups, adopting the significance level *p* = 0.05.

## Results

The results are shown in Table 1 through 5.

**Table 1:** The structure of the population investigated by age, body weight, and BMI

Variable	Average	Median	Min.	Max.	Range	Standard deviation
Age (year)	40.30	35	18	78	60	16.80
Weight (kg)	70.80	71	43	100	57	9
BMI (kg/m^2^)	24.10	23.90	16.20	33.20	16.90	2.80

BMI, body mass index.

Among all surveyed participants, the most numerous age group consisted of people aged 20–35 (47.83%; *n*=287; mean=28.5); the smallest number of people (3.51%; *n*=21) were under 20 years old. The remaining age groups were as follows: 36–50 (18.33%; *n*=110; mean=45.0), 51–65 (18.0%; *n*=108; mean=56.0), and over 65 (12.33%; *n*=74). The average age among all surveyed persons was 40.3 (±16.9 years); the youngest person was 18 and the oldest 78 (Table 1).

The survey showed that milk was most often consumed by the respondents several times a week (70.5% of the total). The percentage of persons consuming milk once or several times a day was only 13.3%. In the surveyed group, there were no people not consuming milk at all. Respondents reported consuming cream with similar frequency, several times a week (69.8%). In the case of fermented milk drinks, both natural and fruit, the highest consumption was two to six times a week (55.0% vs. 58.2%). In addition, for cheese, the respondents most often chose cottage cheese – several times a week (60.0%). Butter was used by 52.0% of respondents several times a day (Table 2). There was no statistically significant relationship between the sex of the surveyed and the choice of the individual variants of answers listed in the table. The relationship is shown between age and consumption of cream (*p*=0.03439). The correlation of variables such as age and consumption of cream is low.

**Table 2:** Frequency of consumption of milk and milk products in the surveyed population

	Daily		2–6 times a week		Less often		Never	
	
	*n*	%	*n*	%	*n*	%	*n*	%
Milk	80	13.30	423	70.50	97	16.20	0	0
Natural milk drinks (e.g. yoghurt)	33	5.50	330	55	217	36.10	20	3.30
Fruit milk drinks (e.g. yoghurt)	174	29	349	58.20	61	10.20	16	2.70
Cheese	62	10.30	287	47.90	231	38.60	20	3.30
Blue cheese	25	4.20	158	26.30	362	60.30	55	9.20
Cream cheese	49	8.2	182	30.30	349	58.20	20	3.30
Cottage cheese	69	11.50	360	60	153	25.50	18	3
Fromage blanc	37	6.1	258	43	277	46.10	28	4.70
Dessert cheese	44	7.30	256	42.60	289	48.20	11	1.80
Butter	312	52	141	23.50	91	15.10	56	9.30
Sour cream	64	10.70	419	69.80	103	17.20	14	2.30

Participants consumed ultra heat treated (UHT) milk most often (61.8%) and pasteurised milk less often (33.3%), whereas the smallest percentage of participants (4.8%) reported consuming raw milk (Table 3). Among all surveyed women, UHT milk was consumed most often (63.1%). The men reported similar consumption of UHT milk (60.1%). In both groups, raw milk was consumed least often. It was consumed by only 5.0% of women and 4.6% of men. In the youngest surveyed group, pasteurised milk was consumed most often (47.6%), whereas in the oldest and other groups UHT milk was most frequently consumed. Most people consuming raw milk were aged 20–35 (44.8%). More than half of the respondents (62.8%) declared that they most often consumed milk with 1–3% fat content. Milk with a fat content of more than 3% was consumed by the smallest percentage of surveyed persons – only 15.0% of the surveyed group. The sex and age of the respondents had no effect on the type of milk most frequently consumed.

**Table 3:** The type of milk consumed, by gender

Gender		UHT milk	Pasteurised milk	Raw milk	Total	Statistics
Male	*n*	157	92	12	261
	%	26.17	15.33	2	43.50
Female	*n*	214	108	17	339	χ^2^*p=*0.67959
	%	35.67	18	2.83	56.50
Total	*n*	371	200	29	600
	%	61.83	33.33	4.83	100

Most respondents (64.8% of the total) thought that milk products were an important part of their diet (medium share). There was no statistically significant relationship between the sex and age of respondents and their answer to the question about the position occupied by milk products in their diet. Among both women and men (Table 4) and in the individual age groups (Table 5), those surveyed declared most often that milk was an important part of their diet (medium share).

**Table 4:** Position of milk products in participants’ diet, by gender

Gender		Small share	Medium share	Big share	Total	Statistics
Male	*n*	48	169	44	261
	%	18.39	64.75	16.86	100
Female	*n*	58	220	61	339	χ^2^*p*=0.88412
	%	17.11	64.90	17.99	100
Total	*n*	106	389	105	600
	%	17.67	64.83	17.50	100

**Table 5:** Position of milk products in participants’ diet, by age

Age group		Small share	Medium share	Big share	Total	Statistics
<20	*n*	1	15	5	21
	%	4.76	71.43	23.81	100
20–35	*n*	57	184	46	287
	%	19.86	64.11	16.03	100
36–50	*n*	19	69	22	110
	%	17.27	62.73	20	100	χ^2^*p*=0.14492
51–65	*n*	14	69	25	108
	%	12.96	63.89	23.15	100
over 65	*n*	15	52	7	74
	%	20.27	70.27	9.46	100
Total	*n*	106	389	105	600
	%	17.67	64.83	17.50	100

Among all those surveyed, milk or milk products were most often consumed between meals (39.5%; *n*=237), for breakfast (35.0%; *n*=210), or for dinner (19.5%; *n*=117), whereas their consumption at lunch was declared by the smallest percentage of people (6.0%; *n*=36).

## Discussion

Our results indicated that the daily requirement of milk and milk products is not being fully met. Only 13% of those surveyed consumed milk on a daily basis; milk was most often consumed a few times a week, as reported by nearly 40% of those surveyed. The respondents, however, used a wide range of milk products. Increased interest in highly processed and potentially more attractive products such as yoghurt, kefir, buttermilk, and cheese has been observed in recent years, both in the domestic market and in the global market (14).

Among fermented milk drinks, products with added fruit were consumed more often than natural ones. This applied to yoghurt in particular; fruit yoghurt was chosen most often (65.67% of the total). Yoghurt with cereals/muesli was chosen by only about 8% of the participants.

The consumption of fermented milk products is recommended for preventing certain kinds of cancer (10). In their study, Larsson et al. noted a substantial reduction in the risk of death from bladder cancer in the Japanese population in people drinking milk and acidophilus milk (15). Cox and Sneyd proved that the regular consumption of milk and milk products in childhood reduces the risk of colon cancer (16). Interesting conclusions have also been drawn by Cho et al. who showed the consumption of ricotta cheese in amounts of more than 25 mg per day to be protective against colon cancer. In the same study, the researchers noted the relationship between drinking more than a glass of milk a day (250 ml) and decreased prevalence of colon cancer, resulting in a risk reduction of 15% (17).

Simultaneously, a meta-analysis of studies on the impact of milk and milk products on increased risk of prostate cancer in men (10 cohort studies, 13 case studies, 2 ecological studies) noted that the results of seven cohort studies showed increased risk of prostate cancer with increased consumption of milk and milk products. (This relationship was statistically significant in two studies only; *p*=0.05.) Two cohort studies showed insignificant reduction in risk and one showed no impact (18). As also indicated by their authors, the results must be interpreted with care. Prior to making modifications to dietary recommendations, it is necessary to conduct studies unequivocally confirming these reports.

One of the most frequently consumed milk products is butter. This product was consumed several times a day by 29% of those surveyed. At the same time, butter was not consumed at all by 9.3% of all respondents.

Butter is a major dietary source of milk fat. As results from numerous studies have shown, milk fat exhibits a multidirectional beneficial effect on the body. It positively affects pro- and antioxidant homeostasis (19). The protective effect of antioxidants found in milk fat on the respiratory tract epithelium has also been demonstrated (20).

Apart from the synergic action of the antioxidants found in milk fat, it is a source of anticancer nutrients including natural trans isomers of fatty acids – vaccenic acid and conjugated linoleic acid (CLA). These acids have universal, health-improving effects: immunostimulating, antioxidant, antisclerotic, and anticancer (21). It has been demonstrated that CLA works multidirectionally: it regulates the lipid profile of the blood and prevents hypertriglyceridemia, obesity, and type 2 diabetes, as well as inflammations (19, 21). The health-improving effect of milk fat on the gastrointestinal tract consists of stimulating the functioning of the intestinal epithelium by short- and medium-chain saturated fatty acids, as well as its ability to bind bacterial toxins and rotaviruses by prostaglandins (21).

Components of milk fat stimulate the functioning of the gastrointestinal tract. It has been demonstrated that saturated fatty acids have an antibacterial effect; therefore, milk fat prevents stomach ulcers and mitigates inflammations of the intestines, *inter alia*, in Lesniowski-Crohn's disease (22). Saturated fatty acids, just like phospholipids, inhibit the growth of *Helicobacter pylori*, reducing the ability of pathogens to survive and colonise the digestive tract (22).

A diet rich in milk products is most frequently promoted for prevention of osteoporotic fractures. Milk contains many nutrients of special significance for bone tissue (4). However, as proven by some authors, increased consumption of milk does not significantly reduce the risk of bone fractures, either in women or men (12).

As shown by some authors, high milk consumption may have undesirable effects related to the presence of D-galactose. Experimental evidence in several species of animals shows that the chronic exposure to D-galactose is harmful to health and causes changes reminiscent of the natural aging process to the body, along with a reduction in vitality due to oxidative stress (23, 24). Meta-analyses of cohort studies do not show, however, a clear structure of such risk and demonstrate that there is no clear evidence from randomised trials (25, 26). It is advisable, however, as emphasised unequivocally by researchers, to separate the consumption of milk from the consumption of other milk products, mainly fermented ones, due to the large difference in the content of lactose and D-galactose. Fermented milk products show the clear induction of oxidative stress (27), possible anti-inflammatory effects (28, 29), and positive impact on the intestinal microbiome (30, 31). High consumption of fermented milk products is related to lower risk of circulatory system diseases (29, 32), whereas high milk consumption is related to the trend of an adverse risk profile for the development of diabetes and circulatory system diseases (29, 33).

Other domestic studies obtained results that were close to those of our own research. Calyniuk et al. (34) found that, of all dairy products, milk was the product consumed most frequently, but not every day. Only about one-third of respondents declared that they had milk every day. Neither our own research nor any other domestic studies have indicated a statistically significant relation between gender and age and milk consumption. The results of our research to assess the frequency of milk consumption agreed with those of studies by other authors that were carried out in different regions of the country (34–36).

All of them confirmed low milk consumption. Insufficient milk consumption was also indicated by the results of research done on younger age groups (37, 38).

Milk and milk products are one of the most valuable food product groups and play a very important role in rational human nutrition, which is responsible for maintaining health. It would be of importance in relation to the presented reports to target consumer preferences to the developing market of milk products and to promote their consumption. Paying attention to suitable amounts of, in particular, fermented milk products in the diet should be an essential element of nutrition education. Appropriate nutrition recommendations may support the prevention and treatment of diseases often caused by improper nutrition.

## Conclusions

On the basis of the studies conducted, the following conclusions may be drawn:

1. The level of consumption of milk and milk products among Upper Silesian agglomeration inhabitants is insufficient in relation to nutrition recommendations. However, despite many controversies surrounding milk, our respondents also claimed that it played an important role in their daily diet.
2. The most frequently consumed type of milk in the surveyed group is UHT milk with average fat content.
